# Chromogen-based double immunohistochemical detection of mitochondrial respiratory chain deficiencies in human brain tissue

**DOI:** 10.1186/s40478-025-01980-7

**Published:** 2025-03-20

**Authors:** Tale L Bjerknes, Anna Rubiolo, Omnia Shadad, Ole-Bjørn Tysnes, Charalampos Tzoulis

**Affiliations:** 1 Neuro-SysMed Center of Excellence for Clinical Research in Neurological Diseases, Department of Neurology, Haukeland University Hospital, Bergen, 5021 Norway; 2 Department of Clinical Medicine, University of Bergen, Bergen, 5020 Norway; 3 K.G. Jebsen Center for Translational Research in Parkinson’s disease, University of Bergen, Bergen, 5020 Norway

**Keywords:** Human brain, Immunohistochemistry, Mitochondria, Mitochondrial respiratory chain

## Abstract

Studies of the mitochondrial respiratory chain (MRC) have given important insights into the pathology of mitochondrial and neurodegenerative disorders. Immunohistochemical methods for staining MRC complexes are particularly valuable for assessing quantitative changes in situ, especially in complex tissues with cellular heterogeneity, such as the brain. However, traditional approaches have notable limitations. Chromogen-based staining, while preserving tissue morphology, has been restricted to a single antigen per section, preventing co-assessment of MRC complexes and mitochondrial mass on the same section. Immunofluorescence, which allows multiplex staining of multiple targets, partially addresses this limitation but compromises tissue morphology and can be highly variable in postmortem brain samples. To address these challenges, we have established a dual-antigen, chromogen-based immunohistochemical method that allows simultaneous assessment of each MRC complex and the mitochondrial marker voltage-dependent anion channel 1 (VDAC1) on the same section. As proof of concept, we apply this method on brain tissue from patients with neurological disease caused by mutations in the mitochondrial DNA polymerase gamma (POLG). Our findings demonstrate that this approach is both reliable and robust. Moreover, this method enables more precise identification of MRC deficiencies in neurons and significantly reduces the amount of tissue required for analysis, a critical advantage when working with scarce human brain samples.

## Introduction

Mitochondrial oxidative phosphorylation (OXPHOS) is a critical process for energy metabolism, and the primary source of cellular adenosine triphosphate (ATP). OXPHOS takes place at the mitochondrial respiratory chain (MRC), which is localized at the inner mitochondrial membrane and comprises five enzyme complexes (complex I to V) and two mobile electron carriers (ubiquinone and cytochrome c) [1]. Quantitative and functional deficiencies of the MRC have been implicated in a wide range of health and disease states, including mitochondrial disorders [2], neurodegenerative diseases such as Parkinson’s disease (PD) [3, 4], Alzheimer’s disease (AD) [5], and amyotrophic lateral sclerosis (ALS) [6], as well as in aging [7, 8]. Given its broad relevance, the development and application of reliable methods to detect and assess MRC deficiencies in post-mortem brain tissue is of paramount importance.

MRC is commonly assessed by quantitative methods, such as immunohistochemistry (IHC) and immunoblotting, and functional measurements of specific complex activities [9–12]. Interpretation of bulk tissue data can be complicated, given that mitochondrial abundance and MRC complex stoichiometry can vary significantly across different cell types, complicating the interpretation of bulk tissue data. On the other hand, IHC is a more cumbersome, low-throughput method, both in execution and interpretation, but offers the unique advantage of identifying changes at the single-cell level. IHC has been extensively used to map MRC complexes across different tissues, including the brain in both mitochondrial and neurodegenerative disorders [6, 13], as well as in aging [14, 15].

Traditionally, IHC of brain tissue has been performed to detect a single antigen at a time. Staining for MRC enzymes and mitochondrial markers has typically been conducted on sequential sections from the same brain region, using horseradish peroxidase (HRP) with the brown substrate 3,3’-diaminobenzidine (DAB) [11, 16], and analysis of stained sections involves manually counting DAB-positive and -negative neurons from digital images. However, identifying negative neurons can be challenging, as their cytoplasm lacks distinct color, leading to potential uncertainty in interpretation. The lack of co-staining for a mitochondrial marker also makes it difficult to know whether regional variations in mitochondrial mass contribute to differences in staining.

To address this limitation, introducing a second mitochondrial antigen, such as VDAC1, stained with a different color would enhance the accuracy of identifying neurons lacking specific MRC complexes. In recent years, a magenta-colored HRP substrate has been described, offering compatibility with automated staining protocols and the ability to be combined with DAB staining [17]. Building on these advancements, we aimed to adapt and validate a sequential double-staining method using DAB and magenta chromogens for quantitative measurements of MRC complexes in human brain tissue. To develop and validate this approach, we used samples from patients with *POLG*-related disease. This disease model is particularly well-suited for validating the double-staining chromogen method, as it exhibits a characteristic profile of MRC deficiencies: specifically, deficiencies in complexes I and IV while sparing complex II [13].

## Methods

### Patient cohorts and tissue samples

Sections were prepared from formalin-fixed, paraffin-embedded tissue samples from frontal cortex, occipital cortex, hippocampus and cerebellum from three individuals with mitochondrial disease caused by mutations in the *POLG* gene, encoding the catalytic subunit of the mitochondrial DNA-polymerase gamma, and three neurologically healthy controls. The areas were selected to provide a comprehensive assessment of the method’s efficacy across different brain regions that exhibit variable susceptibility to complex I deficiency. The individuals carrying the *POLG* mutations had a clinical phenotype of spinocerebellar ataxia, external ophtalmoplegia, epilepsy, stroke-like episodes and peripheral neuropathy. The genotypes and demographic and clinical characteristics of the subjects are summarized in Table 1.

**Table 1:** Demographic information

Patient	Mutation	Sex	Age at Onset (years)	Age at Death (years)	PMI (hours)
POLG1	A467T/A467T	F	15	44	48
POLG2	A467T/A467T	M	8	47	NA
POLG3	W748S/W748S	F	17	43	24
CONTROL1		F		95	30
CONTROL2		F		69	33
CONTROL3		F		80	34.5

PMI: post-mortem interval

### Immunohistochemical staining procedure

Serial 3.5 μm thick sections were stained using primary antibodies against MRC complex I (NDUFS4 antibody, Abcam ab137064, dilution 1:500), complex II (SDHA antibody, Abcam ab14715, dilution 1:1000) and complex IV (MTCOI antibody, Invitrogen 459600, dilution 1:3000), visualized by MACH4 Universal HRP-polymer (Biocare M4U534) and DAB chromogen kit (Biocare DB801), before sequential staining with the mitochondrial marker porin (VDAC1 antibody, Abcam ab14734, dilution 1:10000), visualized by MACH 4 Universal HRP-polymer (Biocare M4U534) and Magenta (Agilent, GV900). Tacha’s haematoxylin (Biocare, NM-HEM-M) was used as counterstain.

Slides were prepared for automated staining by using the deparaffinization and antigen target retrieval Dako PT-link from Agilent with the low pH EnVision FLEX Target Retrieval Solution at 98 °C for 24 min. A protocol for automated staining using the above substrates was made for the Autostainer Link 48 from Agilent Technologies, adapted from a previously published protocol for DAB/magenta sequential double staining [17] (Table 2). Briefly, endogenous peroxidase was blocked by hydrogen peroxide (Peroxidase 1, Biocare, PX968M), before incubation with the primary antibodies against either complex I, II or IV visualized by HRP and DAB. To prevent cross-reactivity between reagents, an acidic block using sulfuric acid was applied to dissociate the antibodies from their antigens so that they could be washed off before the next staining round as previously described [17, 18]. After the sulfuric acid step, the IHC cycle was again repeated with VDAC1 as the primary antibody visualized by HRP and Magenta. After completion of the automated staining procedure, sections were dehydrated in graded alcohol (increasing steps from 70% to 100%) and Xylene, before coverslipping.

**Table 2:** Staining protocol for autostainer link 48

Step	Category	Reagent	Incubation time (min)
1	Rinse	TBS Automation wash buffer	0 min
2	Endogenous enzyme block	Peroxidazed 1	5 min
3	Rinse	DI water	0 min
4	Rinse	TBS Automation wash buffer	5 min
5	Primary antibody	Primary antibody placeholder	30 min
6	Rinse	DI water	0 min
7	Rinse	TBS Automation wash buffer	5 min
8	Secondary reagent*	Mouse Linker	15 min
9	Rinse*	DI water	0 min
10	Rinse*	TBS Automation wash buffer	5 min
11	Labelled polymer	HRP polymer	30 min
12	Rinse	DI water	0 min
13	Rinse	TBS Automation wash buffer	5 min
14	Substrate-chromogen	DAB Biocare	8 min
15	Rinse	DI water	0 min
16	Rinse	TBS Automation wash buffer	5 min
17	Enzyme block	Sulfuric acid	5 min
18	Rinse	TBS Automation wash buffer	0 min
19	Rinse	Water	5 min
20	Rinse	TBS Automation wash buffer	5 min
21	Endogenous enzyme block	Peroxidazed 1	5 min
22	Rinse	DI water	0 min
23	Rinse	TBS Automation wash buffer	5 min
24	Primary antibody	VDAC1 antibody	30 min
25	Rinse	DI water	0 min
26	Rinse	TBS Automation wash buffer	5 min
27	Secondary reagent	Mouse Linker	15 min
28	Rinse	DI water	0 min
29	Rinse	TBS Automation wash buffer	5 min
30	Labelled polymer	HRP polymer	30 min
31	Rinse	DI water	0 min
32	Rinse	TBS Automation wash buffer	5 min
33	Substrate-chromogen	HRP-Magenta	5 min
34	Rinse	DI water	0 min
35	Rinse	TBS Automation wash buffer	5 min
36	Counterstain	Tacha’s Haematoxylin	3 min
37	Rinse	DI water	0 min
38	Rinse	DI water	5 min
39	Rinse	TBS Automation wash buffer	0 min

* Steps included for SDHA and MTCOI antibodies

### Image analyses

Slides were scanned digitally using NanoZoomer XR (Hamamatsu) microscope. Positive and negative neurons were visually evaluated by two independent investigators (TLB and AR) using the NDP.view2plus version 2.7.25 software (Hamamatsu). Areas of 3.5 mm^2^-4 mm^2^ spanning cortical layers 2–6 were evaluated from the prefrontal cortex and occipital cortex, whereas all neurons in the CA4 area of the hippocampus and all Purkinje cells in the cerebellar slides were evaluated. Slides were evaluated at a 40X magnification.

## Results

**Fig. 1:**
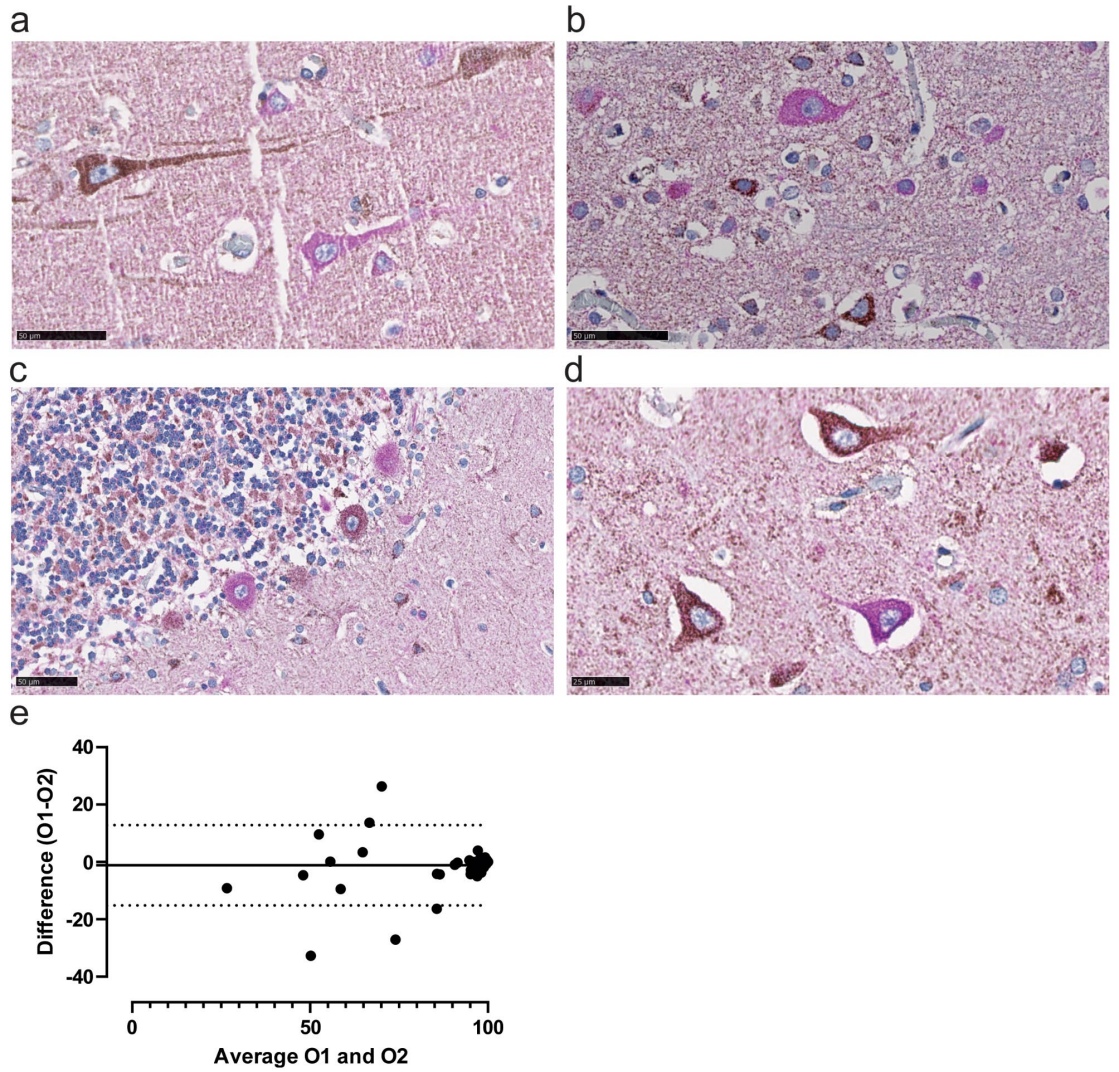
Double Chromogen Staining of NDUFS4 and VDAC1. (**a**-**d**) Representative images of neurons from POLG tissue staining positive for NDUFS4, MRC complex I, characterized by a brown-stained cytoplasm, or negative for NDUFS4, exhibiting a magenta-stained cytoplasm. The magenta staining corresponds to VDAC1, a marker of mitochondrial mass, while the DAB brown chromogen indicates the presence of mitochondrial respiratory complex proteins (in this case, NDUFS4). Figures are from(a) prefrontal cortex, (**b**) occipital cortex (**c**) cerebellum, and (**d**) CA4 region of the hippocampus. Scale bars: 50 μm (**e**) Bland–Altman plot comparing measurements between Observer 1 (O1) and Observer 2 (O2). The x-axis represents the mean of the measurements from both observers, while the y-axis shows the difference between them

Technically, the double immunostaining worked well and consistently. There was no obvious influence of postmortem interval for MRC complexes I, II or IV (Table 1). Positive neurons for complexes I, II and IV were identified by brown DAB-staining in the cytoplasm, whereas negative neurons were identified as having only magenta colored cytoplasm, indicating single staining of the mitochondrial marker VDAC1 (Fig. 1a, b,c, d). The sharp contrast between positive and negative neurons allowed for a fast and efficient quantitative assessment of the proportion of positive neurons per section, with a high inter-rater reliability (ICC coefficient = 0.91, 95% C.I. [0.851–0.942], *p* < 0.001). This reliability was further supported from the Bland-Altman analysis, showing a small mean difference between observers with narrow limits of agreement (Fig. 1e). As expected, inter-rater reliability was highest when the percentage of positively stained neurons approached 100%, likely reflecting the inherent challenge of visually assessing intermediate staining levels, where subjective interpretation plays a greater role. Nevertheless, overall agreement remained strong, as evidenced by the high ICC value (Fig. 1e).

**Fig. 2:**
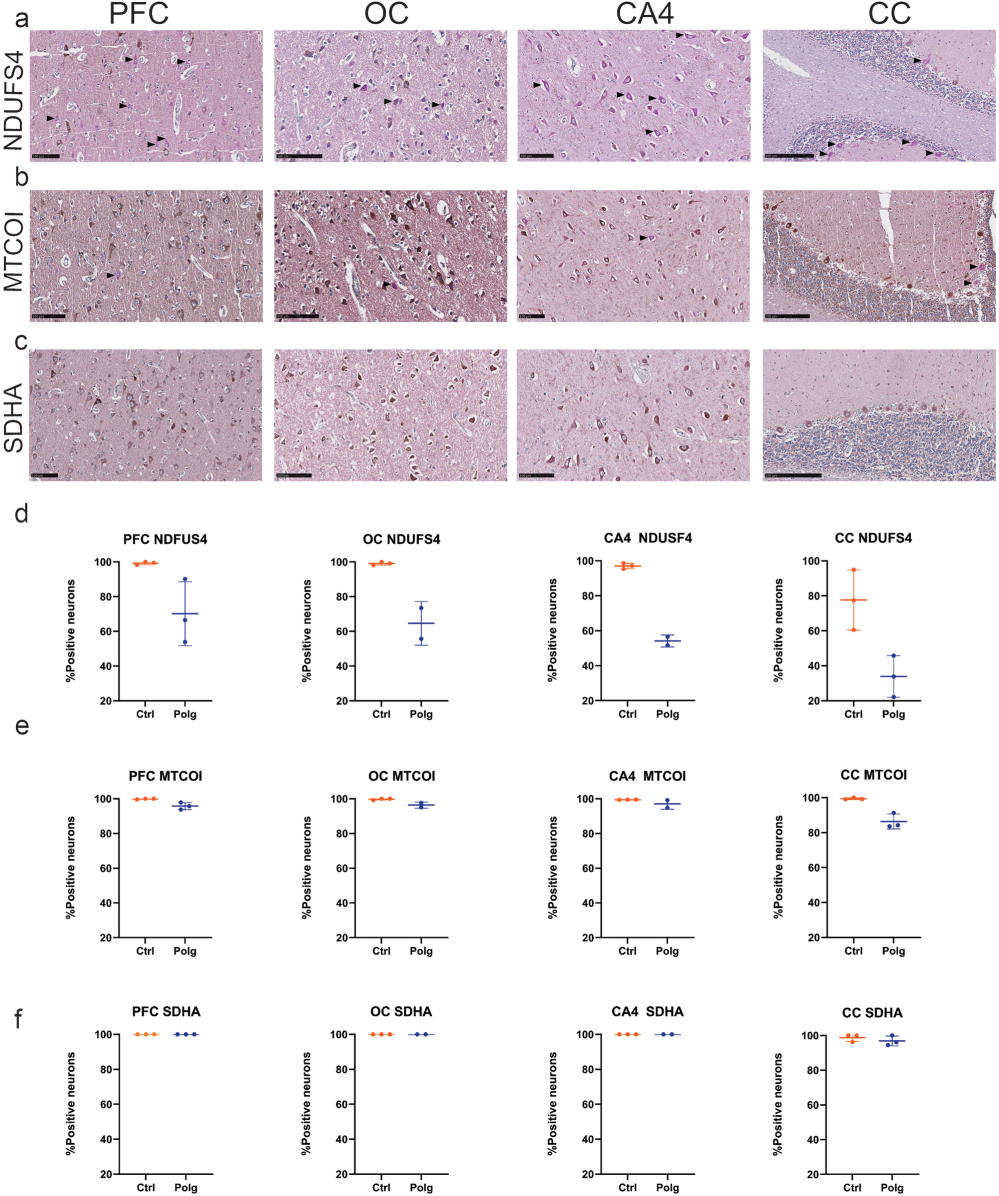
Mitochondrial respiratory complex staining across brain regions in *POLG* disease and controls. Representative images show double chromogen immunohistochemical staining for (**a**) NDUFS4, (**b**) MTCOI, and (**c**) SDHA in the prefrontal cortex (PFC), occipital cortex (OC), CA4 region of the hippocampus, and cerebellar cortex (CC) of individuals with POLG-disease. Black arrows indicate neurons negative for MRC complexes I, II, or IV, but positive for VDAC1. Scale bars: 100 μm for PFC, OC, and CA4; 250 μm for CC. (**d**–**f**) Scatterplots showing the percentage of neurons positive for (**d**) NDUFS4, (**e**) MTCOI, and (**f**) SDHA in the same brain regions of individuals with POLG-disease (POLG, blue) and controls (Ctrl, orange). Scatterplots show individual values (dots), median and interquartile range

NDUFS4-immunostaining revealed a prominent reduction in the percentage of complex I positive neurons across all examined brain regions of individuals with *POLG*-disease, compared to controls (Fig. 2a, d; Table 3). The most profound deficiency was observed in the Purkinje cells of the cerebellum (Mean ± SD: *POLG*: 33.90% ± 11.85; Control: 77.60% ± 17.23) and the hippocampal CA4 region (Mean ± SD: *POLG*: 54.14% ± 3.46; Control: 97.03% ± 1.61). The prefrontal and occipital cortex also exhibited a noticeable reduction in the percentage of NDUFS4-positive neurons (Mean ± SD PFC: *POLG*: 70.10 ± 18.39; Control: 99.26% ± 0.88; Mean ± SD OC: *POLG*: 64.63% ± 12.59; Control: 99.10% ± 0.92), albeit to a lesser extent.

**Table 3:** Descriptive statistic IHC for NDUFS4

NDUFS4 + VDAC1	Complex I positive neurons, %									
	Observer 1				Observer 2					
	PFC	OC	CA4	CC	PFC		OC	CA4	CC	
POLG1	90.14	55.73	NA	22.08	91.17		55.64	NA	31.21	
POLG2	53.91	NA	51.69	45.78	63.33		NA	47.69	50.41	
POLG3	66.52	73.53	56.58	33.85	63.12		59.88	57	66.52	
Mean ± SD	70.10 ± 18.39	64.63 ± 12.59	54.14 ± 3.46	33.90 ± 11.85	72.54 ± 16.13		57.76 ± 2.99	52.35 ± 6.58	49.38 ± 17.68	
Control1	100	98.16	95.39	94.92	99.69		98.77	97.83	97.75	
Control2	99.5	99.13	97.08	60.47	99.35		99.90	99.25	87.55	
Control3	98.25	100	98.61	77.42	97.24		99.61	99.68	93.79	
Mean ± SD	99.26 ± 0.88	99.10 ± 0.92	97.03 ± 1.61	77.60 ± 17.23	98.76 ± 1.33		99.43 ± 0.59	98.92 ± 0.97	93.03 ± 5.14	

PFC: prefrontal cortex; OC: occipital cortex; CC: cerebellar cortex

MTCO1 staining, which indicates the presence of complex IV, revealed a mild yet consistent deficiency across all four brain regions in *POLG* tissues compared to control samples (Fig. 2b, e; Table 4). Although the decrease in MTCO1-positive neurons was less pronounced than that seen with complex I, there was still a notable reduction, especially in Purkinje cells (Mean ± SD: *POLG*: 86.39% ± 4.26, Control: 99.42% ± 0.50). The hippocampal area CA4, the prefrontal and occipital cortices also exhibited a mild reduction in MTCO1 staining, reflecting the partial deficiency in complex IV commonly associated with *POLG*-related mitochondrial disorders (Mean ± SD CA4: *POLG*: 97% ± SD; Control: 99.47% ± 0.01). PFC: *POLG*: 95.80% ± 2.06; Control: 99.87% ± 0.23; Mean ± SD OC: *POLG*: 96.35% ± 1.77; Control: 99.75% ± 0.43).

**Table 4:** Descriptive statistic IHC for MTCOI

MTCOI + VDAC1	Complex IV positive neurons, %								
	Observer 1				Observer 2				
	PFC	OC	CA4	CC	PFC		OC	CA4	CC
POLG1	97.89	97.60	NA	84.26	99.81		99.27	NA	88.61
POLG2	93.77	NA	99.14	83.61	96.64		NA	97.30	87.79
POLG3	95.74	95.09	94.86	91.30	95.74		94.55	98.21	91.57
Mean **±** SD	95.80 ± 2.06	96.35 ± 1.77	97.00 ± 3.03	86.39 ± 4.26	97.40 ± 2.14		96.61 ± 3.34	97.76 ± 0.64	89.32 ± 1.99
Control1	99.60	99.26	99.46	99.16	99.82		99.67	98.82	95.18
Control2	100	100	99.47	99.11	100		100	98.42	100
Control3	100	100	99.47	99.42	99.71		99.90	99.73	99.61
Mean **±** SD	99.87 ± 0.23	99.75 ± 0.43	99.47 ± 0.01	99.42 ± 0.50	99.84 ± 0.15		99.86 ± 0.17	98.99 ± 0.67	98.26 ± 2.68

In contrast to the deficiencies observed in complexes I and IV, immunostaining for the complex II subunit SDHA showed no difference between individuals with *POLG*-disease and controls in any of the brain regions examined (Fig. 2c, f; Table 5). No neurons were found to be negative for VDAC1 in any of the brain regions examined.

**Table 5:** Descriptive statistic IHC for SDHA

SDHA + VDAC1	Complex II positive neurons, %							
	Observer 1				Observer 2			
	PFC	OC	CA4	CC	PFC	OC	CA4	CC
POLG1	100	100	NA	100	100	100	NA	100
POLG2	100	NA	100	94.51	100	NA	100	99.50
POLG3	100	100	100	96.09	100	99.82	100	96.08
Mean ± SD	100 ± 0	100 ± 0	100 ± 0	96.87 ± 2.82	100 ± 0	99.91 ± 0.13	100 ± 0	98.53 ± 2.13
Control1	100	100	100	100	100	100	100	100
Control2	100	100	100	100	100	100	100	100
Control3	100	100	100	96.09	100	100	100	100
Mean ± SD	100 ± 0	100 ± 0	100 ± 0	98.75 ± 2.17	100 ± 0	100 ± 0	100 ± 0	100 ± 0

## Discussion

Our proof-of-concept results in *POLG*-disease recapitulate previous results in the same individuals, showing severe complex I and milder complex IV defects in cerebellar Purkinje cells, followed by the hippocampal CA4 region, and the cerebral cortex [13]. The observation of a moderate complex I deficiency in the cerebellum of controls is likely related to the higher age of these individuals and corroborates previous findings indicating Purkinje cells are particularly susceptible to age-dependent loss of complex I [19]. No change was observed in the exclusively nuclear encoded complex II. This is in line with the pathogenesis of *POLG*-disease which is driven by the accumulation of mtDNA damage, including multiple deletions, depletion, and point mutations, caused by the mutant DNA-polymerase [13].

The double chromogen approach has several advantages over existing approaches for IHC-based MRC assessment. First, it enables the simultaneous evaluation of MRC complexes and mitochondrial mass within the same cells. This ensures that observed differences in staining intensities are attributed to variations in MRC complex levels, rather than differences in mitochondrial mass per cell. Second, the hematoxylin counterstaining preserves the underlying tissue architecture, a feature that is not achievable with immunofluorescence techniques. Third, the ability to detect two antigens simultaneously minimizes the amount of tissue required for analysis. This is particularly critical in studies of human brain tissue, which is a highly valuable and limited resource. Lastly, the use of DAB and magenta chromogens presents distinct advantages over previous double-IHC methods, such as those combining alkaline phosphatase and HRP with Fast Red and DAB [20]. Fast Red has several drawbacks, including a narrow 30-minute window to use, partial reactivity with the DAB leading to darker staining, and solubility in ethanol and mounting media. Other efforts using a double chromogen approach include the reddish-brown HRP substrate 3-amino-9-ethylcarbazole (AEC) together with the brown DAB-stain [21]. However, this combination is not possible to use for co-localizing targets due to poor color contrast between the substrates. Other approaches for visual assessment of multiple IHC targets include using chromogen removal with sequential staining and digital microscopy to create pseudo-fluorescent images of IHC stained tissue [22, 23]. Blue HRP substrates have also been described as providing good contrast to the DAB stain; however, they do not pair well with the blue hematoxylin nuclear stain [22, 24]. These limitations make the HRP-magenta system a more reliable and effective choice [17].

## Conclusion

Sequential double IHC staining using DAB and magenta chromogens is a reliable and effective method for assessing MRC complexes and mitochondrial mass in individual cells in brain tissue, while preserving tissue morphology. The method offers a valuable contribution to studies of MRC complex deficiencies in human brain disorders.

## Data Availability

The dataset used and analyzed during the current study is available from the corresponding author on reasonable request.

## Abbreviations

MRC: Mitochondrial respiratory chain
VDAC1: Voltage-dependent anion channel 1
POLG: DNA polymerase gamma
OXPHOS: Oxidative phosphorylation
ATP: Adenosine triphosphate
PD: Parkinson’s disease
AD: Alzheimer’s disease
ALS: Amyotrophic lateral sclerosis
IHC: Immunohistochemistry
HRP: Horseradish peroxidase
DAB: 3,3’-diaminobenzidine
NDUFS4: NADH: ubiquinone oxidoreductase subunit S4
SDHA: Succinate dehydrogenase complex flavoprotein subunit A
MTCO1: Mitochondrially encoded cytochrome c oxidase I
AEC: 3-amino-9-ethylcarbazole
